# Use of dual-energy X-ray absorptiometry to evaluate variation in bone shape and alignment associated with radiographic knee osteoarthritis: Findings from a study of 19,053 individuals in UK Biobank

**DOI:** 10.1016/j.ocarto.2025.100667

**Published:** 2025-08-21

**Authors:** Rhona A. Beynon, Faten Alomar, Fiona R. Saunders, Raja Ebsim, Benjamin G. Faber, Mijin Jung, Jennifer S. Gregory, Claudia Lindner, Simon G.F. Abram, Richard M. Aspden, Nicholas C. Harvey, Timothy Cootes, Jonathan H. Tobias

**Affiliations:** a University of Bristol, Musculoskeletal Research Unit, Bristol Medical School, Bristol, United Kingdom; b University of Aberdeen, Centre for Arthritis and Musculoskeletal Health, Aberdeen, United Kingdom; c The University of Manchester, Division of Informatics, Imaging & Data Sciences, School of Health Sciences, Manchester, United Kingdom; d University of Bristol, Medical Research Council Integrative Epidemiology Unit, Bristol, United Kingdom; e University of Southampton, MRC Lifecourse Epidemiology Centre, Southampton, United Kingdom; f NIHR Southampton Biomedical Research Centre, University of Southampton and University Hospital Southampton NHS Foundation Trust, United Kingdom

**Keywords:** Knee osteoarthritis, Dual-energy X-ray absorptiometry, Statistical shape modelling, Knee alignment

## Abstract

**Objective:**

Lower limb alignment may predispose to, or exacerbate, symptoms of knee osteoarthritis. To examine the role of this and other joint shape variation, we conducted a cross-sectional study investigating relationships between radiographic knee osteoarthritis (rKOA) and knee shape in dual-energy X-ray absorptiometry (DXA) images from UK Biobank (UKB).

**Methods:**

Associations between the first ten knee shape modes (KSMs), derived from statistical shape modelling, and rKOA grade were analysed using logistic regression, adjusting for age, sex, height, weight, and ethnicity. An additional model included adjustment for hip-knee-ankle (HKA) angle, derived from total body DXA scans, to reflect knee alignment. Composite figures illustrate knee shape characteristics associated with each rKOA grade.

**Results:**

19,053 individuals were included (mean 63.7 years, 48 ​% males), of whom 80.7 ​%, 14.6 ​%, 3.6 ​% and 1.2 ​% had rKOA grades 0, 1, 2 and 3–4, respectively. Several KSMs were associated with rKOA in confounder-adjusted analyses, with higher grades showing stronger relationships. These associations were attenuated by adjustment for HKA. As expected, composite shape models revealed that higher rKOA grades were associated with greater varus malalignment. After HKA adjustment, composite shape models showed less varus alignment, with other shape differences, such as altered proximal tibial metaphysis and lateral patella displacement, emerging in higher-grade rKOA.

**Conclusions:**

Our cross-sectional analyses between joint shape and DXA-derived rKOA grade showed expected relationships with varus malalignment, which were attenuated after adjusting for HKA. Other shape differences, particularly in higher-grade rKOA, emerged independently of alignment, warranting further investigation.

## 1 Introduction

Knee osteoarthritis (KOA) is a common and debilitating condition characterised by the progressive degeneration of articular cartilage, alongside changes in the subchondral bone, synovial membrane, and surrounding soft tissues [1]. Malalignment of the limb alignment, such as varus (bow-legged) and valgus (knock-kneed), often worsens with the progression of KOA and frequently exacerbates symptoms associated with mechanical overload in the affected knee compartment(s) [2]. Varus alignment places increased stress on the medial compartment of the knee, while valgus alignment places greater stress on the lateral compartment [3]. Lower limb alignment is a significant predictor of KOA severity and progression, including radiographic worsening (e.g. increased joint space narrowing (JSN) and/or higher Kellgren-Lawrence (KL) grades, functional decline, and increased pain [[2], [3], [4]]. Variation in lower limb alignment has also been associated with disease onset in some [[3], [4], [5]], but not all studies [6].

While varus and valgus deformities have been widely studied, other aspects of knee shape may also influence the development and progression of KOA. Statistical shape modelling (SSM) has emerged as a valuable tool for quantifying complex, multidimensional variations in bone shape by identifying modes of variation—key patterns of shape differences among individuals [7]. SSM has been used in various studies to evaluate overall hip shape and examine how its variation relates to pathologies such as hip osteoarthritis [8]. For example, in a recent study using UK Biobank (UKB) data, we used machine learning techniques to analyse the relationships between radiographic hip osteoarthritis (rHOA), derived from dual-energy X-ray Absorptiometry (DXA) images, and joint shape, as evaluated by SSM. Moderate rHOA was associated with femoral neck widening and increased acetabular coverage, while severe rHOA exhibited cam morphology and reduced acetabular coverage [9]. Some of this shape variation may reflect a hypertrophic sub-type of hip osteoarthritis characterised by excessive bone formation [10]; however, whether the same occurs at the knee is currently unknown.

Applying a similar approach to the knee could uncover shape variations beyond coronal plane lower limb alignment, providing insights into how variations in knee shape contribute to KOA progression and outcomes such as total knee replacement. We recently derived radiographic knee osteoarthritis (rKOA) measures from approximately 20,000 right knee DXA images in the UKB and confirmed expected relationships with subsequent risk of joint replacement [11]. We also applied SSM to examine knee joint shape in individuals who later underwent joint replacement, identifying shape patterns consistent with varus deformity [12]. However, it remains unclear whether the observed association between knee shape and KOA risk is due to knee alignment alone, or if other aspects of knee shape also contribute to KOA severity.

This study aims to examine the relationship between knee shape, as determined by SSM applied to DXA images from UKB, and varying severities of KOA. Additionally, it seeks to determine whether these associations remain after adjusting for hip-knee-ankle (HKA) angle, a commonly used radiographic measure of knee alignment [13], which we have recently derived using a novel machine learning approach applied to total body DXA scans. A better understanding of how knee shape influences KOA progression may support the development of targeted interventions focused on specific morphological features, potentially slowing disease progression.

## 2 Methods

### 2.1 Study population

UKB is a large prospective cohort study that has gathered phenotypic and genetic data from around 500,000 individuals across the UK, who were between 40 and 69 years old at the time of their recruitment (2006–2010). As part of the Imaging Enhancement Study, a subset of this cohort underwent imaging, including knee and total body DXA scans. DXA scans were performed in a supine position using a high-resolution iDXA scanner (iDXA GE-Lunar, Madison, WI, USA). Height and weight were measured at the time of the DXA scan using standardized procedures, while age, sex, and ethnicity were recorded via a touchscreen questionnaire during the same assessment visit. The UKB Ethics Advisory Committee provides ethical oversight and guidance for the entire project, including this study under application 17295. All participants gave informed consent before taking part in the study.

### 2.2 SSM

**Fig. 1:**
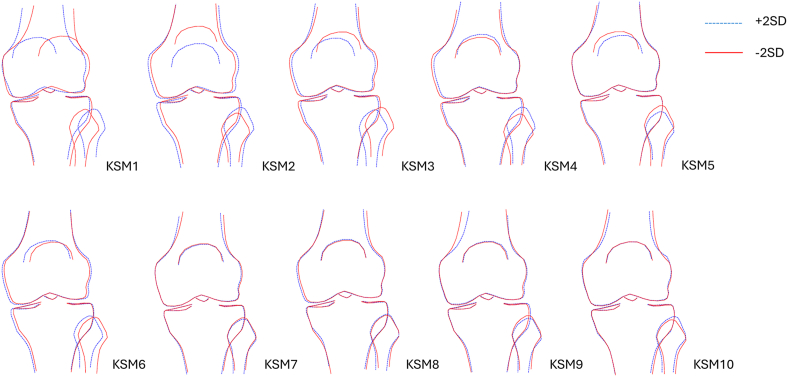
First 10 KSMs derived from knee DXA scans. Shapes +2 standard deviations (SD) from the mean are shown in blue, whilst shapes -2SD from the mean are shown in red.

A 129-point SSM was previously developed using left knee DXA scans [12]. Briefly, an automated search model (BoneFinder®, The University of Manchester) was trained on ∼7000 manually annotated left knee DXA images to delineate the distal femur, proximal tibia, proximal fibula, and superior patella. This model was applied to an additional ∼31,000 left knee DXA images. The images were visually inspected by two trained annotators (RB, FS), and the automated point placements were manually adjusted as necessary. After point placement, Procrustes analysis was performed to align points, followed by principal component analysis to construct the SSM from all available images. This process generated a set of orthogonal modes of shape variation, referred to as knee shape modes (KSMs). Each individual received a score for each KSM, representing how many standard deviations their knee shape deviated from the mean. Collectively, these KSMs explained 100 ​% of the variance in the dataset. Subsequent analyses focused on the first 10 KSMs, which accounted for 79.5 ​% of the shape variance (see Fig. 1). The variance explained by each additional KSM was minimal, with no individual mode contributing more than 2 ​%. The automated search model and knee SSM have since been applied to all available right knee DXA scans, with around 20,000 images manually verified to ensure accurate point placement before application of the SSM.

### 2.3 DXA-based radiological evaluation

The rKOA grades were determined based on previously established methods [11]. These methods involved manual annotation of osteophytes on all 20,000 right knee DXA images reviewed for point placement, along with the automated measurement of the minimum joint space width (mJSW). Briefly, osteophyte grades (0–3) were determined at four sites on the medial and lateral femur and tibia, based on specific cut-offs of manually shaded osteophyte area (mm^2^). The mJSW of the medial joint compartment (mm) was measured between specific outline points on the femoral and tibial plateaus and used to derive medial JSN grades. JSN grades were defined as follows: grade 0 for mJSW ≥3 ​mm, grade 1 for mJSW ≥2.5 ​mm and <3 ​mm, grade 2 for mJSW ≥2 ​mm and <2.5 ​mm, and grade 3 for mJSW <2 ​mm. Because the total osteophyte sum across all four sites could equal 12 – considerably higher than the maximum JSN grade of 3 – the osteophyte grades were weighted by 0.5 before summation, resulting in a combined osteophyte score ranging from 0 to 6. The weighted osteophyte score was then added to the JSN grade to generate the overall rKOA score (0–9), which was subsequently grouped into rKOA grades (0–4).

### 2.4 Ascertainment of knee alignment

**Fig. 2:**
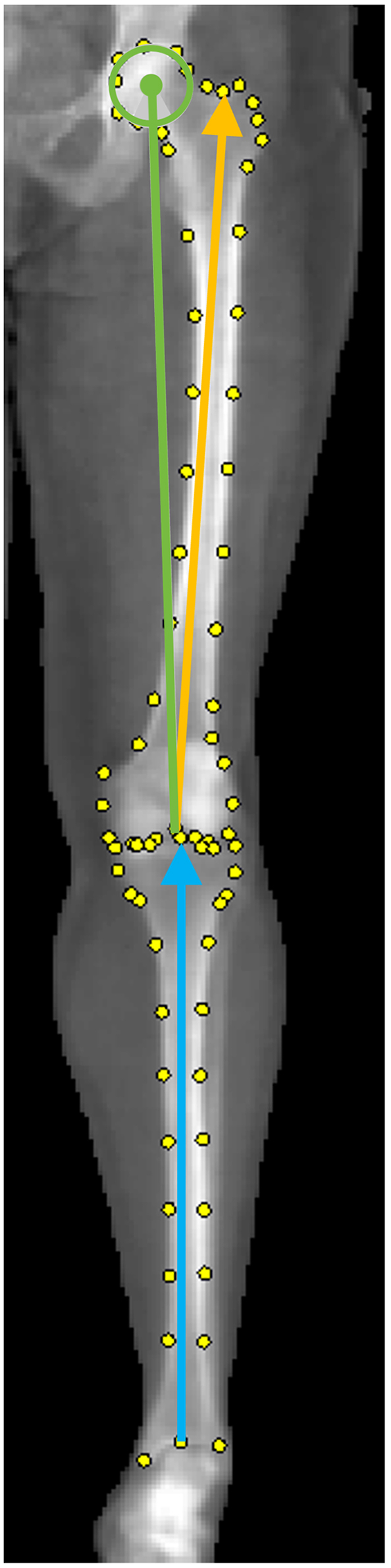
Landmarks used to calculate alignment measures from total body DXA scans. The tibial shaft axis (TA, blue arrow) extends from the ankle to the knee centre (intercondylar notch). The mechanical femur axis (FA, green line) runs from the centre of the femoral head to the centre of the knee joint. The hip–knee–ankle (HKA) angle is defined as the angle between the TA and the mechanical FA, while the femorotibial angle (FTA) is defined as the angle between the TA and the anatomical FA (orange arrow).

Mechanical and anatomical lower limb alignment were measured by deriving HKA and femorotibial angle (FTA) respectively on total body DXA scans obtained at the same time as the knee scans, with HKA being our primary measure of interest. These geometric measurements were based on specific anatomical landmarks automatically identified by the BoneFinder® software. Specifically, the femoral head centre was calculated by fitting a circle to seven landmarks around the femur head. HKA was calculated as the angle formed by the intersection of the mechanical axes of the femur (the line from femoral head centre to the landmark at the centre of the femoral intercondylar notch) and the tibial shaft axis (the line from the landmark at the centre of the tibial plateau to the centre of the talus) (Fig. 2). FTA was calculated as the angle formed by the intersection of the anatomical axis of the femur (the line connecting the centre of the femoral shaft) and the tibial shaft axis.

### 2.5 Statistical analysis

Descriptive statistics are reported as means and ranges for continuous variables and as absolute and relative frequencies (%) for categorical variables. To examine the relationships between the first 10 KSMs and rKOA grade, separate binary logistic regression models were conducted for each grade, using grade 0 (no rKOA) as the reference category. For each rKOA grade, three models were employed: Model 1 - unadjusted model, Model 2 - adjusted for demographics including age, sex, height, weight, and ethnic category, and Model 3 - additionally adjusted for HKA angle. To assess possible effect modification by sex, we included a sex-by-KSM interaction term in the adjusted models. Both unadjusted and adjusted odds ratios (OR) with 95 ​% confidence intervals (CI) are presented. The ORs reflect changes per 1 standard deviation (SD) increase in KSM. A Bonferroni-corrected significance threshold of 0.005 (0.05/10 KSMs) was used to account for the 10 independent KSM exposures tested. All statistical analyses were performed using Stata (StataCorp. 2023. Stata Statistical Software: Release 18. College Station, TX: StataCorp LLC). To illustrate the overall impact of knee shape on rKOA, we constructed a composite shape vector for each rKOA grade by weighting each of the first 10 KSMs using the confounder-adjusted beta coefficients from the fully adjusted logistic regression models, with each mode scaled by its corresponding unstandardised SD from the original KSM distribution. For visualization, an arbitrary scaling of five was applied before combining values into a single vector, showing the shape change associated with increasing rKOA grade.

## 3 Results

### 3.1 Population characteristics

At the time of these analyses, 19,159 right knee DXA scans with both knee shape data and rKOA classification were available after applying quality control measures. These measures included the removal of low-quality images, those with metal artifacts obscuring the bone border, extreme rotation, or insufficient tibial or femoral shaft length, as well as participant withdrawals. From this dataset, HKA angle could be derived in 19,053 individuals, after removing cases where the search on the whole-body images failed due to poor image quality, incomplete images, significant hip abnormality, hip implants or metal artifacts. The mean age of participants was 63.7 years (SD 7.5) (52 ​% females) (Table 1). 15,377 (80.7 ​%) of participants were rKOA grade 0, 2775 (14.6 ​%) of participants had rKOA grade 1681 (3.6 ​%) rKOA grade 2152 (0.80 ​%) rKOA grade 3 and 68 (0.4 ​%) rKOA grade 4. Those with rKOA grades 3 and 4 were combined in subsequent analyses, due to the smaller number of individuals in these categories, which is expected in a relatively young population, to ensure sufficient statistical power.

**Table 1:** Baseline descriptive statistics of the study population.

	Total	Female	Male
	N ​= ​19053	N ​= ​9864	N ​= ​9189
	Mean (SD)		
Age (years)	63.72 (7.53)	63.01 (7.42)	64.49 (7.58)
Height (cm)	170.20 (9.46)	163.59 (6.45)	177.29 (6.61)
Weight (kg)	75.18 (15.05)	67.89 (12.78)	83.02 (13.27)
Ethnic background	N		
White	18443 (96.80)	9560 (96.92​)	8883 (96.67)
Asian	202 (1.06)	83 (0.84​)	119 (1.30)
Chinese	51 (0.27)	34 (0.34)	17 (0.19)
Black	113 (0.59)	61 (0.62)	52 (0.57)
Mixed	87 (0.46)	47 (0.48)	40 (0.44)
Other ethnic group	107 (0.56)	58 (0.59)	49 (0.53)
Unknown	50 (0.26)	21 (0.21)	29 (0.32)
Radiographic measures			
rKOA grade	N		
0	15377 (80.71)	7315 (74.16)	8062 (87.74)
1	2775 (14.56)	1956 (19.83)	819 (8.91)
2	681 (3.57)	474 (4.81)	207 (2.25)
3	152 (0.80)	84 (0.85)	68 (0.74)
4	68 (0.36)	35 (0.35)	33 (0.36)
	Mean (range)		
FTA	4.90 (−14.47, 17.21)	5.70 (−12.77, 17.21)	4.04 (−14.47, 14.03)
HKA	−0.62 (−18.74, 11.46)	0.21 (−15.42, 11.46)	−1.51 (−18.74, 7.91)

### 3.2 Association of individual KSMs with rKOA grade

**Fig. 3:**
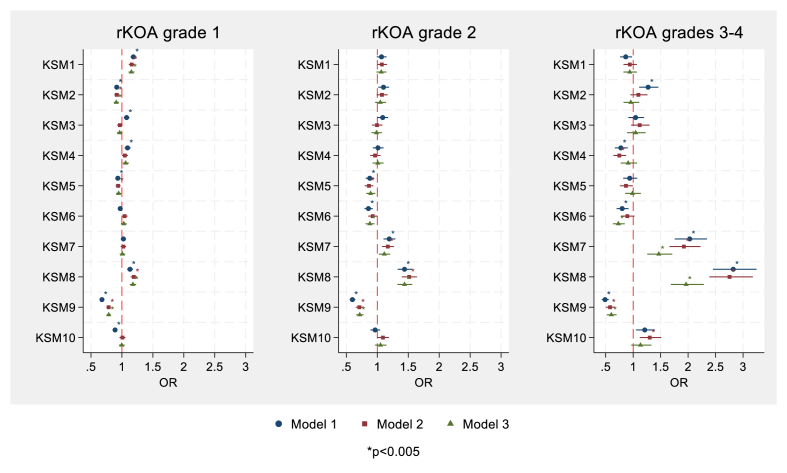
Associations of KSMs with rKOA grades in combined sex analyses. Results show OR with 95 ​% confidence intervals, per SD increase in KSM. Model 1: unadjusted; Model 2: adjusted for age, sex, height, weight and ethnic group; Model 3: additionally adjusted for hip-knee-ankle (HKA) angle. Abbreviations: OR, odds ratio; KSM, knee shape mode; rKOA, radiographic knee osteoarthritis.

Fig. 3 displays the OR and 96 ​% CIs for associations between the first 10 KSMs and varying grades of rKOA in the combined-sex analyses, with numerical results provided in Supplementary Table 1). Each OR reflects the change in odds of a given rKOA per 1 SD increase in the corresponding KSM. Results are shown both unadjusted and adjusted for demographic factors (age, sex, height, weight, and ethnicity). Sex-stratified analyses are shown in Supplementary Figs. 1–2 and Supplementary Tables 2–3. Illustrations of the 10 KSMs are provided in Fig. 1, with accompanying descriptions available in Supplementary Table 5 of Beynon et al. [12].

In the combined-sex analysis adjusted for demographic factors, five KSMs showed strong associations with rKOA grade 1. Specifically, increases in KSMs 1 and 8 were associated with higher odds of grade 1 rKOA (KSM1 OR ​= ​1.16; 95 ​% CI: 1.11–1.21; KSM8 OR ​= ​1.19; 95 ​% CI: 1.14–1.23), while increases in KSMs 2, 5, and 9 were associated with lower odds (KSM2 OR ​= ​0.91; 95 ​% CI: 0.87–0.96; KSM5 OR ​= ​0.93; 95 ​% CI: 0.90–0.98; KSM9 OR ​= ​0.78; 95 ​% CI: 0.75–0.82). When interaction terms between sex and each KSM were included in the adjusted models, KSMs 7 and 9 demonstrated evidence of sex-based effect modification, indicated by asterisks in Supplementary Table 1. As shown in sex-stratified analyses, this reflected a positive association with KSM7 in males but not females, and a stronger negative association with KSM9 in males compared to females (Supplementary Figs. 1–2; Supplementary Tables 2–3).

For rKOA grade 2, after adjustment for demographic factors, increases in KSM 5 and KSM 7 were associated with lower and higher odds of rKOA grade 2 respectively, in sex-combined-sex analyses (OR ​= ​0.87; 95 ​% CI: 0.80–0.94 for KSM 5, and OR ​= ​1.18; 95 ​% CI: 1.10–1.28 for KSM 7). Associations were also seen for KSMs 8 and 9, with stronger relationships than those observed for rKOA grade 1 (OR ​= ​1.52; 95 ​% CI: 1.40–1.65 and OR ​= ​0.71; 95 ​% CI: 0.65–0.77 for KSMs 8 and 9, respectively). The association between KSM5 and rKOA grade 2 showed evidence of a sex interaction, with a negative association seen in males but not females in sex-stratified analyses.

Stronger associations, which are more likely to be clinically meaningful, were observed between KSMs and rKOA grades 3–4, in sex-combined analyses adjusted for demographic factors. KSM4 was inversely associated with rKOA grades 3–4 (OR ​= ​0.76; 95 ​% CI:0.645–0.88). KSMs 7, 8, and 9 were more strongly associated with rKOA grades 3–4, compared to lower grades (combined analysis: KSM7 OR ​= ​1.94; 95 ​% CI: 1.67–2.25; KSM8 OR ​= ​2.78; 95 ​% CI: 2.40–3.20; KSM9 OR ​= ​0.58; 95 ​% CI: 0.50–0.67). Additionally, KSM10 showed a positive association with rKOA grades 3–4 (OR ​= ​1.31; 95 ​% CI: 1.13–1.52). The association between KSM4 and rKOA grades 3–4 showed evidence of a sex interaction, sex-stratified analyses revealing a negative association in females but not males.

### 3.3 HKA-adjusted associations

To assess whether the associations between KSMs and rKOA grades persisted after accounting for knee alignment, additional models were run adjusting for HKA angle. After this adjustment, the relationships between KSMs 8 and 9 and rKOA grades 1, 2 and 3–4 remained in both combined (Supplementary Table 1) and sex-stratified analyses (Supplementary Tables 2 and 3), although the effect estimates were partially attenuated (see Fig. 3). KSMs 1 and 2 continued to be associated with rKOA grade 1 in the combined analysis and in females, with similar effect estimates. KSM 7 was associated with rKOA grades 3–4 in both combined and female analyses, although, the strength of this association was reduced compared to models that adjusted only for demographic characteristics. Additionally, after adjusting for HKA, KSM 6 was inversely associated with rKOA grade 2 in the combined analysis, and with grades 3–4 in both the combined analysis and in females. Similar results were obtained after adjusting for FTA angle (Supplementary Tables 1–3 for combined, male and female analyses). Sex-interactions broadly mirrored those seen in analyses only adjusted for demographic factors (see Supplementary Table 1).

### 3.4 Composite knee shape models

**Fig. 4:**
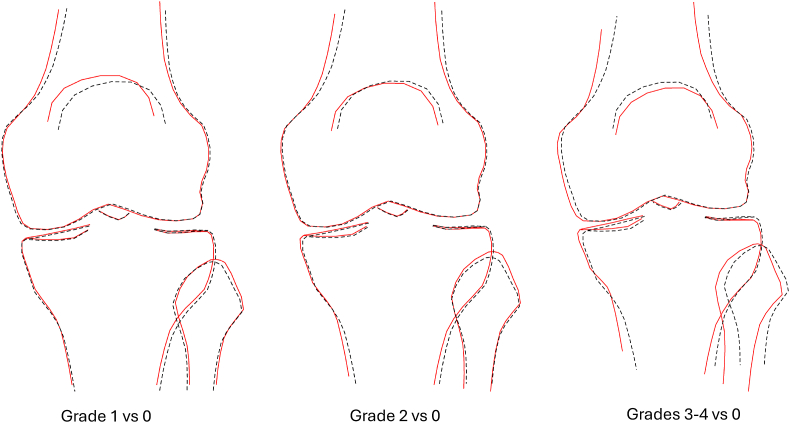
Composite models adjusted for age, sex, height, weight, and ethnicity (sex combined). Dashed black line ​= ​normal healthy knee, red line ​= ​shape associated with rKOA. All shapes are aligned at point 17 of the SSM template, located at the distolateral corner of the femur.

**Fig. 5:**
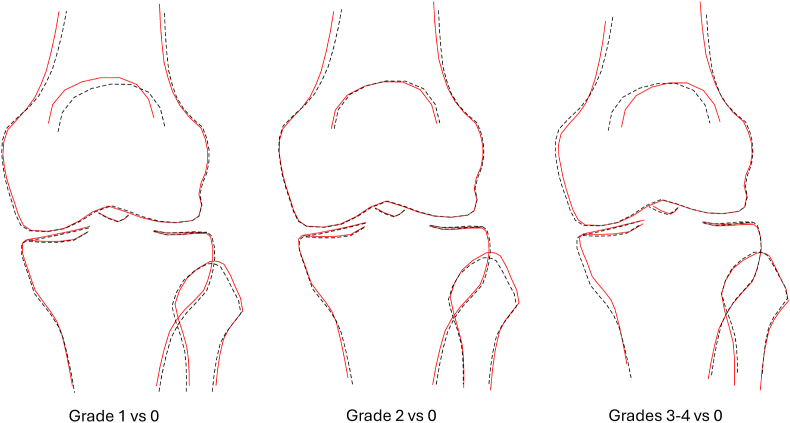
Composite models adjusted for age, sex, height, weight, ethnicity and HKA (sex combined). Dashed black line ​= ​normal, red line ​= ​shape associated with rKOA. Shapes are aligned at point 17 on the SSM template.

KSM associations were combined to visualise the overall knee shape associated with each rKOA grade. In composite models adjusted for demographic factors, the combined sex results showed a progressive shift towards varus alignment with increasing severity of rKOA (Fig. 4). Additionally, participants with grade 3–4 rKOA exhibited a visible reduction in the width of the mm, as well as a widened femoral metaphysis. Similar results were seen in equivalent analyses in males (Supplementary Fig. 3) and females (Supplementary Fig. 4). In the case of composite models further adjusted for HKA, as expected, varus alignment was considerably less marked in relation to rKOA (Fig. 5). Furthermore, there was no longer any suggestion of medial JSN or a widened femoral metaphysis in those with grade 3–4 rKOA. On the other hand, grade 3–4 rKOA showed the appearance of a laterally deviated patella, and a narrower tibial metaphysis. Similar differences were seen in equivalent analyses in males (Supplementary Fig. 5) and females (Supplementary Fig. 6).

## 4 Discussion

We examined associations between whole knee shape as assessed by SSM, and rKOA in approximately 20,000 right knee DXA images from UKB. Inspection of composite shapes derived from combining associations with the first ten KSMs revealed that higher rKOA grades were associated with greater degrees of varus deformity. To examine whether rKOA is also associated with shape changes unrelated to varus deformity, we investigated the effect of adjustment for lower limb alignment. The latter was based on HKA derived from total body DXA scans. Following this further adjustment, the association between rKOA and varus deformity was greatly attenuated. There was no indication of bony expansion akin to that seen in hip osteoarthritis [9]. That said, several KSMs showed associations with rKOA in the fully adjusted model, and inspection of composite shapes revealed other changes which may be of pathological significance, including a laterally deviated patella, and a narrower tibial metaphysis.

The finding of greater varus alignment with increasing grades of rKOA is consistent with the recognised varus malalignment characteristic in KOA [3,4]. This alignment may serve as an important predisposing or exacerbating factor, focusing weight-bearing forces through the medial compartment, resulting in considerable biomechanical stress. Varus alignment may also be a consequence of medial JSN, which was evident in participants with grade 3–4 rKOA. These findings are consistent with our recent study investigating associations between knee shape and risk of subsequent total knee replacement in 37,000 individuals, using DXA-derived measurements from UKB and the same method as described here [12]. The B-score identified in that study, reflecting the shape difference between those with and without subsequent knee replacement, represents similar characteristics to those observed in relation to rKOA in the present analysis after adjusting for demographics, namely varus alignment, medial JSN and widening of the femoral metaphysis.

To establish whether rKOA is related to shape variation other than altered alignment, we developed and applied a separate method for evaluating knee alignment based on total body DXA scans, which were also obtained in UKB participants. This method has the advantage of evaluating knee alignment based on the position of the joints above and below the knee. We evaluated both functional and anatomical alignment through measurement of HKA and FTA respectively [14], use of which gave similar results when adjusting associations with knee shape. Unsurprisingly, adjustment for alignment using this approach largely attenuated the appearance of varus alignment associated with rKOA. This adjustment also attenuated the association of medial JSN with rKOA, confirming that a strongly correlation between varus alignment and medial JSN in the most common phenotypic pattern of rKOA. In future work, other phenotypical patterns of rKOA such as lateral compartment or patellofemoral compartment dominant patterns could be investigated using similar methods that take account of the ‘pre-disease’ joint space measurements.

As for other aspects of shape variation, adjustment for limb alignment led to loss of the association between rKOA and enlargement of the femoral metaphysis. If anything, the femoral metaphysis appeared relatively smaller in those with grade 3–4 rKOA in the fully adjusted analyses. Conceivably, the apparent femoral enlargement observed in analyses unadjusted for alignment may represent some kind of artefact arising from the two-dimensional projection of the image using this method, rather than a true structural change. On the other hand, a similar appearance in the form of widening of the femoral condyles in knees affected by osteoarthritis has also been reported in MRI-based SSM studies based on three-dimensional images [15].

In fully adjusted analyses, grade 3–4 rKOA was also associated with a narrower tibial metaphysis. This could potentially be related to projection due to differences in rotation, in light of findings from a previous CT study that external tibial torsion is reduced in patients with knee osteoarthritis [16]. There was no evidence of shape variation equivalent to cam-type changes suggested to develop as part of advanced hip osteoarthritis [9]. Grade 3–4 rKOA was also associated with a laterally deviated patella in this model.

Two previous studies have used SSM to characterise knee shape in rKOA. Wise et al. analysed relationships between sex, incident KOA and knee shape on plain radiographs of 304 participants from the Osteoarthritis Initiative [17]. Since separate SSM models were generated for the hip and knee, it was difficult to investigate relationships with joint alignment as in the present study. Gregory et al. compared differences in knee shape derived by SSM from iDXA scans in 109 individuals over 12 months [18]. The changes observed predominantly reflected those related to osteoarthritis such as osteophytes rather than separate aspects of joint shape. There were also indications that joint alignment changed over the year alongside medial osteophytes and loss of joint space, however neither HKA nor FTA could be calculated to confirm this, as no total body scans were taken. In addition, the scale of the present study provides the opportunity to identify genetic influences on knee shape, and to use Mendelian Randomisation to examine causal relationships with disease outcomes, as recently undertaken at the hip [19].

In the present study, we aimed to distinguish changes related to joint shape and osteoarthritis by separately annotating bone shape and osteophytes, adjusting reference points to exclude osteophytes where appropriate. That said, any association between rKOA and medial JSN may have arisen since JSN was used in the definition of rKOA. In terms of other limitations, there are some disadvantages in using DXA scans to annotate rKOA. Unlike X-rays, DXA images are acquired with participants in a supine position, as opposed to weight bearing, which typically results in larger measurements of mJSW, although comparative studies remain limited [20,21]. One small study (n ​= ​213) found that the difference in medial mJSW between non-weight-bearing and weight-bearing radiographs were more pronounced for knees with KL grades 2 and 3, while differences were minimal for KL-1 and KL-4 [21]. To our knowledge, no studies have directly compared knee DXA scans to standing weight-bearing radiographs. Therefore, future large-scale longitudinal studies are needed to evaluate how measurement differences between non-weight-bearing and weight-wearing modalities influence KOA classification and prediction. Like X-rays, being two-dimensional, DXA scans also provide a limited view of osteophytes and can be distorted by minor changes in patient positioning, potentially obscuring osteophytes from view. Finally, the generalisability of our findings may be limited, as the UKB cohort is largely composed of white participants (95 ​%) and has a lower all-cause mortality rate compared to the general population, as has been noted previously [22].

In conclusion, having examined associations between knee shape and risk of KOA on DXA images in nearly 20,000 participants from UKB, we found that those with higher grades of rKOA showed evidence of varus deformity, medial JSN and a wider femoral metaphysis. These relationships were largely attenuated after adjusting associations for lower limb alignment. In our fully adjusted model, those with rKOA showed no evidence of bony expansion, and indeed if anything femoral and tibial metaphyses appeared slightly narrower in those with high grade rKOA. Further studies are justified to examine the role of changes in knee shape in rKOA that appear to be independent of malalignment.

## Author contributions

All authors made substantial contributions to the analysis and interpretation of data. RAB, JHT, and BGF contributed to the conception and design of the study, while RAB, FRS, RE, FA, BGF, MJ, and NCH were involved in data acquisition. RAB and JHT prepared the initial draft of the manuscript. All authors critically reviewed the content and approved the final version for submission.

## Role of funding source

This research was conducted using the UKB Resource (application number 17295). It was funded in whole, or in part, by the 10.13039/100010269Wellcome Trust [Grant numbers: 209233/Z/17/Z, 223267/Z/21/Z]. BGF is funded by an 10.13039/501100000272NIHR Academic Clinical Lectureship and an 10.13039/501100000691Academy of Medical Sciences Starter Grant (SGL030∖1057). CL was funded by a Sir Henry Dale Fellowship jointly funded by the 10.13039/100010269Wellcome Trust and the 10.13039/501100000288Royal Society (223267/Z/21/Z). This research was funded in whole, or in part, by the 10.13039/100010269Wellcome Trust [Grant number 223267/Z/21/Z]. For the purpose of open access, the author has applied a CC BY public copyright licence to any Author Accepted Manuscript version arising from this submission. NCH is funded by the 10.13039/501100000265UK Medical Research Council (10.13039/501100000265MRC) [MC_PC_21003; MC_PC_21001], and 10.13039/501100022419NIHR Southampton Biomedical Research Centre, 10.13039/501100000739University of Southampton and 10.13039/100010417University Hospital Southampton NHS Foundation Trust, UK.

## Declaration of competing interest

No competing financial interests exist.
