# Supplementary material for: Use of dual-energy X-ray absorptiometry to evaluate variation in bone shape and alignment associated with radiographic knee osteoarthritis: Findings from a study of 19,053 individuals in UK Biobank
Source: Osteoarthr Cartil Open. 2025 Aug 21;7(4):100667. doi: 10.1016/j.ocarto.2025.100667 (PMC12445559; doi:10.1016/j.ocarto.2025.100667)
Supplement: Multimedia component 1 [file mmc1.docx]

Supplementary Figure 1: Association of KSMs with rKOA grades in males.

Associations of knee shape modes (KSMs) with rKOA grades in males. Results show odds ratios with 95% confidence intervals, per SD increase in KSM. Model 1: unadjusted; Model 2: adjusted for age, height, weight and ethnic group; Model 3: additionally adjusted for hip-knee-ankle (HKA) angle. Abbreviations: OR, odds ratio; rKOA, radiographic knee osteoarthritis.

Supplementary Figure 2: Association of KSMs with rKOA grades in females.

Associations of knee shape modes (KSMs) with rKOA grades in females. Results show odds ratios with 95% confidence intervals, per SD increase in KSM. Model 1: unadjusted; Model 2: adjusted for age, height, weight and ethnic group; Model 3: additionally adjusted for hip-knee-ankle (HKA) angle. Abbreviations: OR, odds ratio; rKOA, radiographic knee osteoarthritis.

Supplementary Figure 3: Male Model 2- adjusted for demographics (age, sex, height, weight, ethnicity).

Grade 1 vs 0

Grade 2 vs 0

Grades 3-4 vs 0


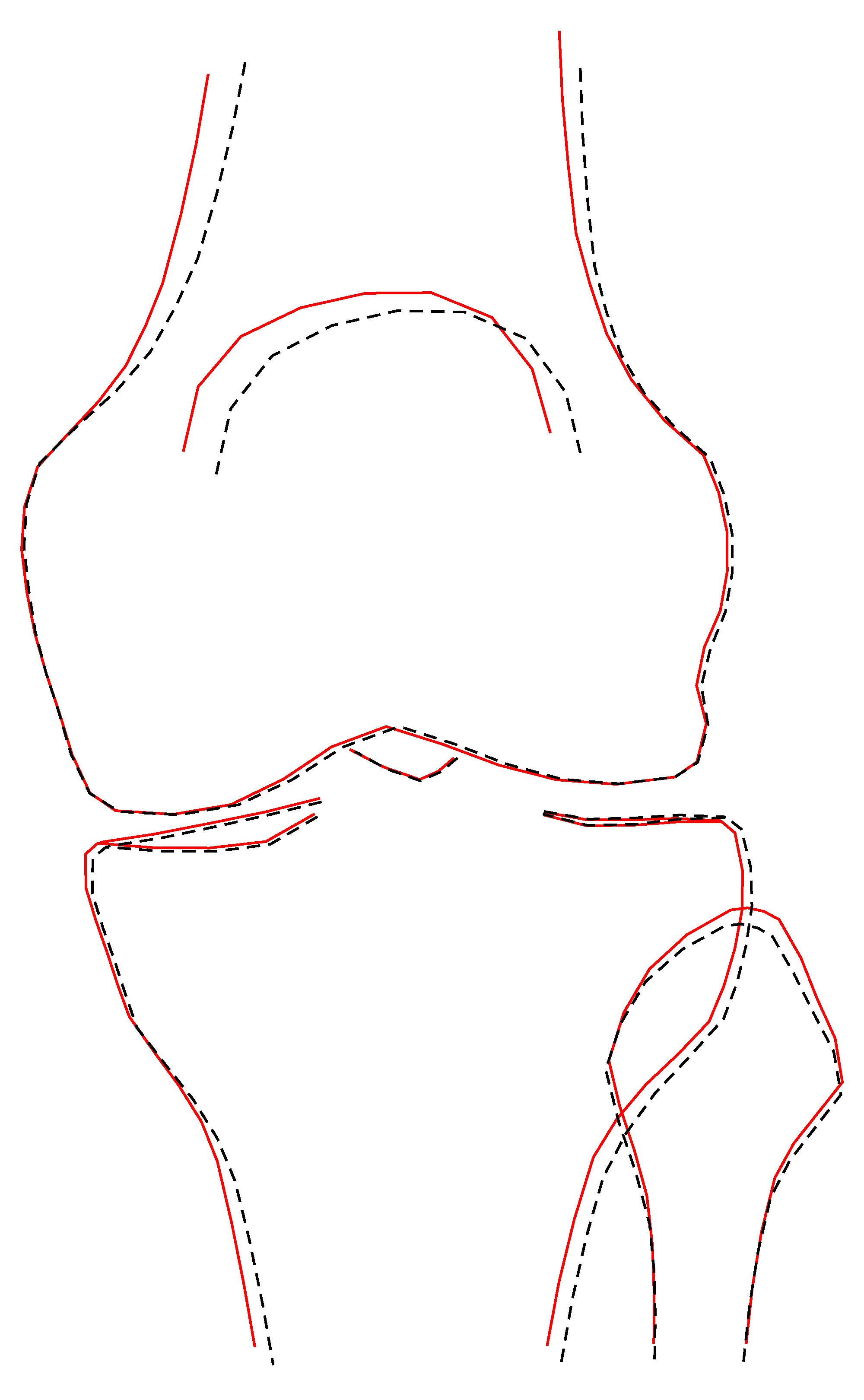

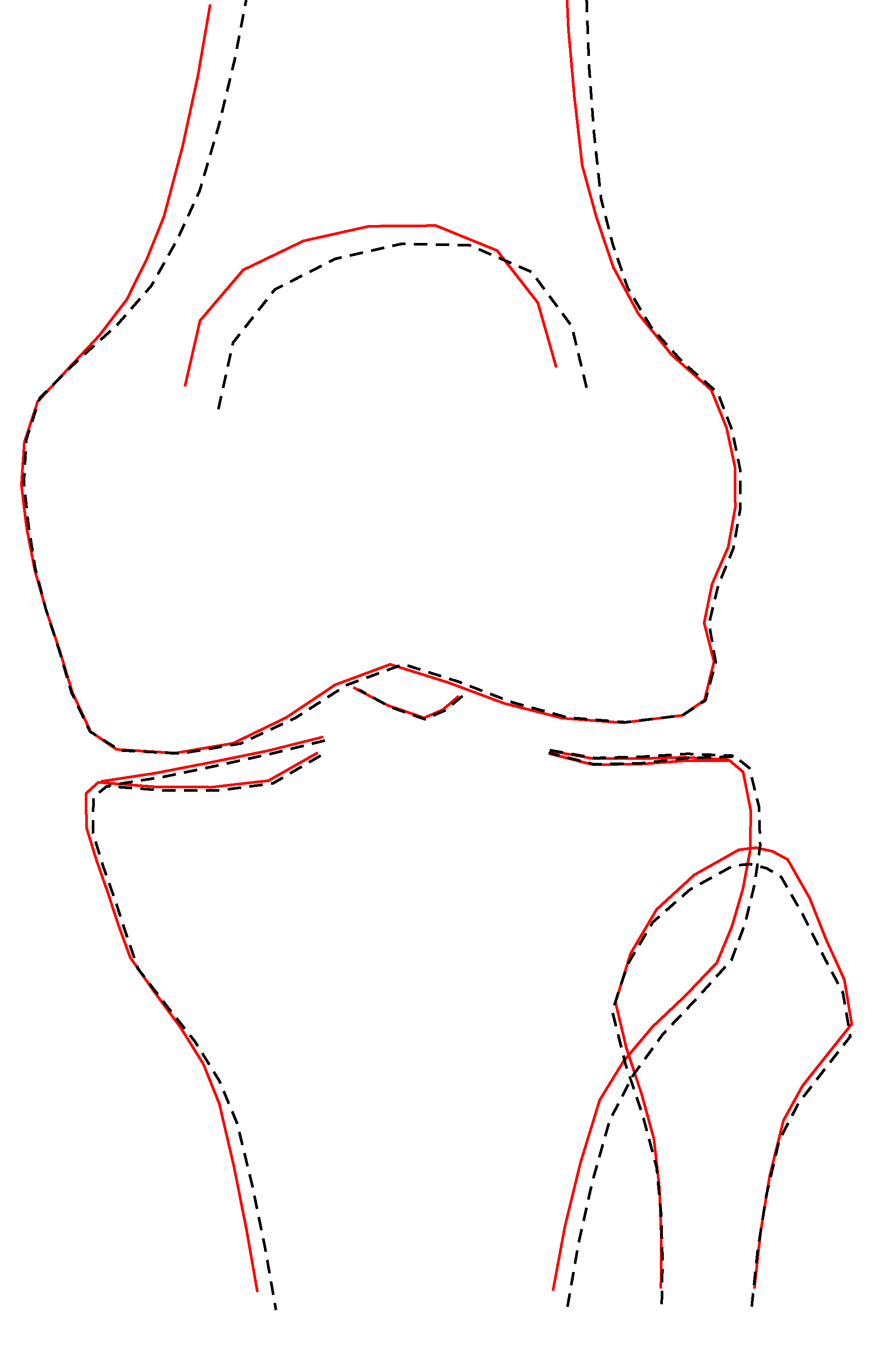

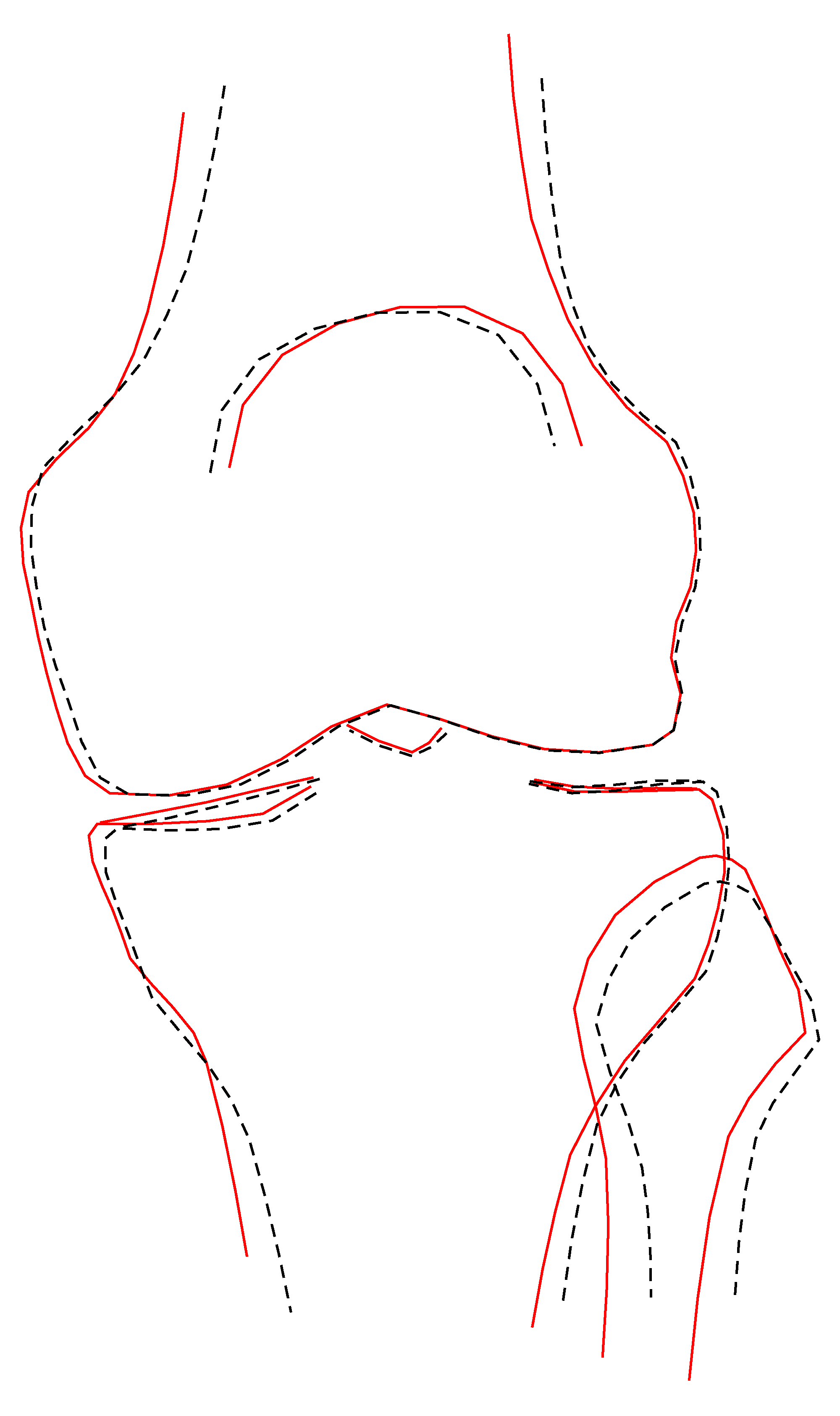


Shapes are aligned at point 17 on the SSM template (corner of the lateral femur).

Shapes are aligned at point 17 on the SSM template (corner of the lateral femur).

Supplementary Figure 4: Female Model 2- adjusted for demographics (age, sex, height, weight, ethnicity)

Grade 1 vs 0

Grade 2 vs 0

Grades 3-4 vs 0


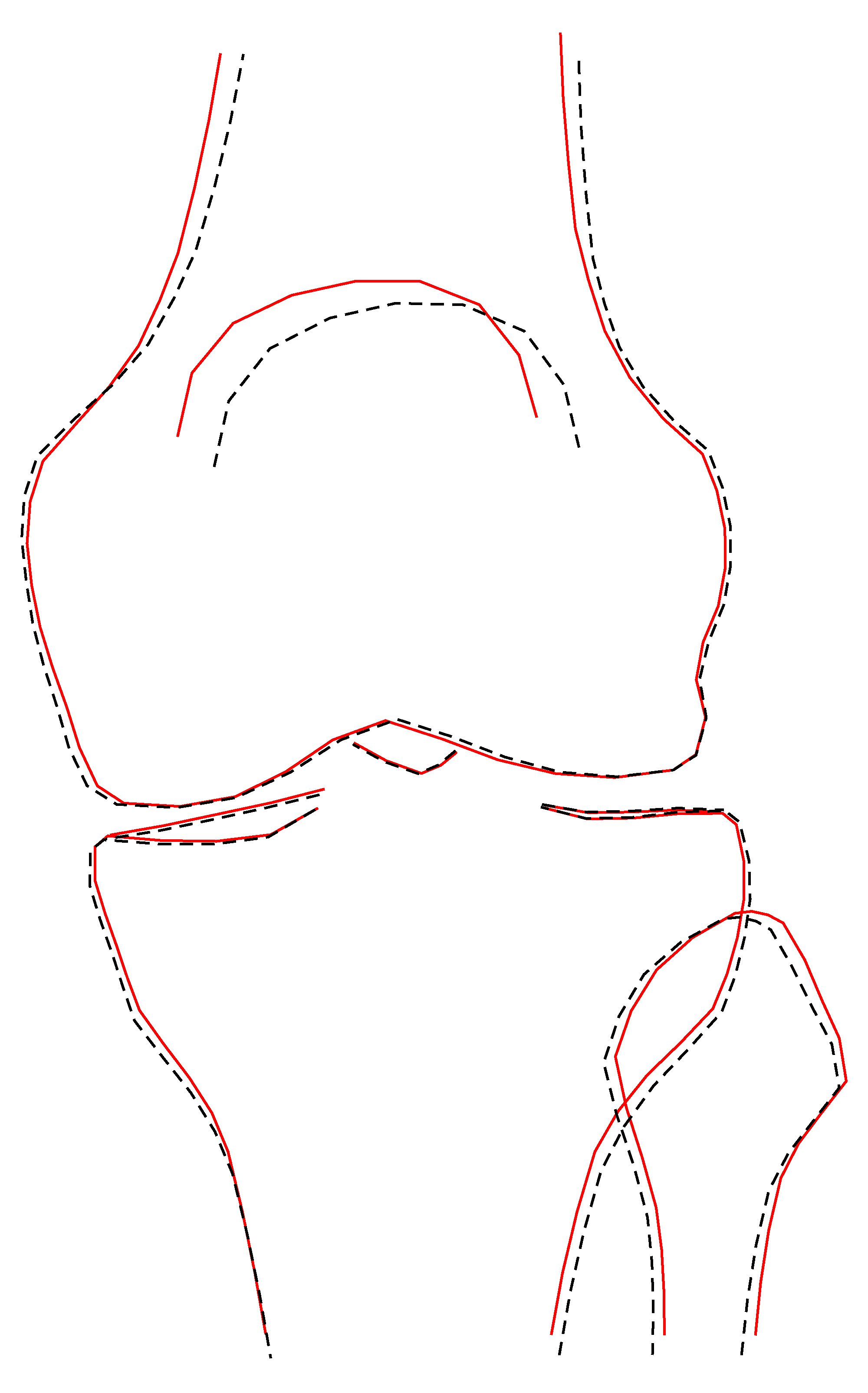

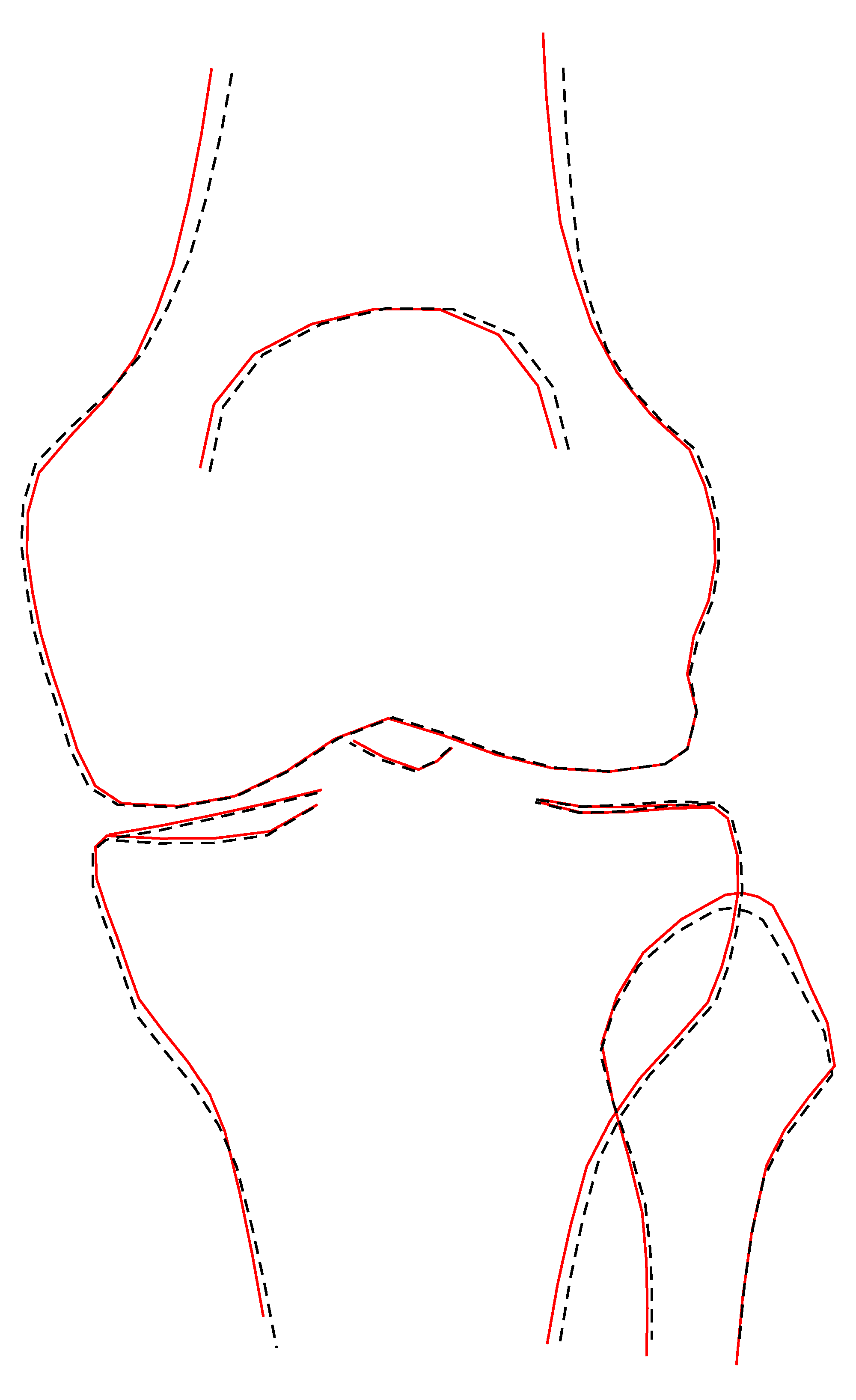

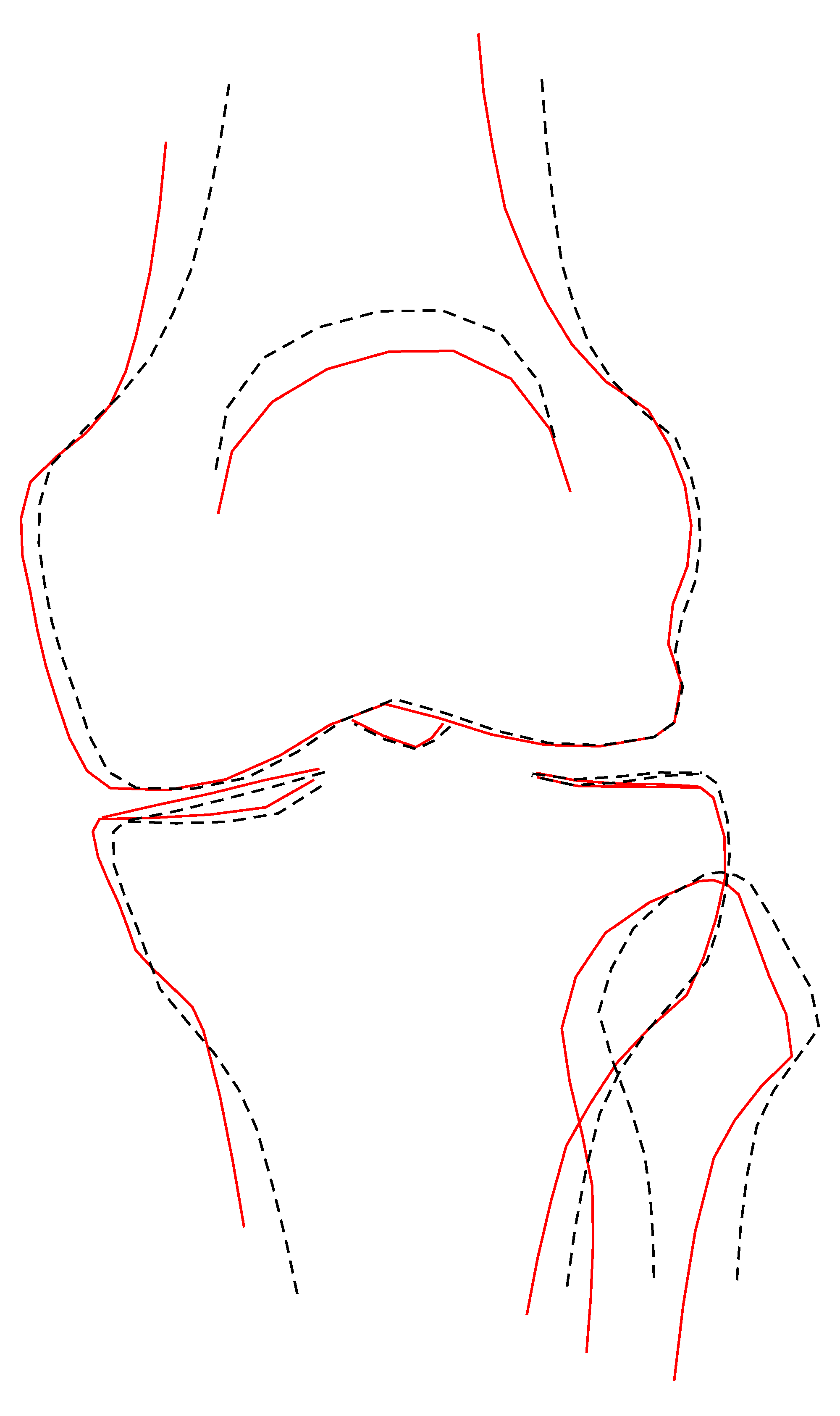


Supplementary Figure 5: Model 3- adjusted for demographics + HKA angle (male)

Grade 1 vs 0

Grade 2 vs 0

Grades 3-4 vs 0


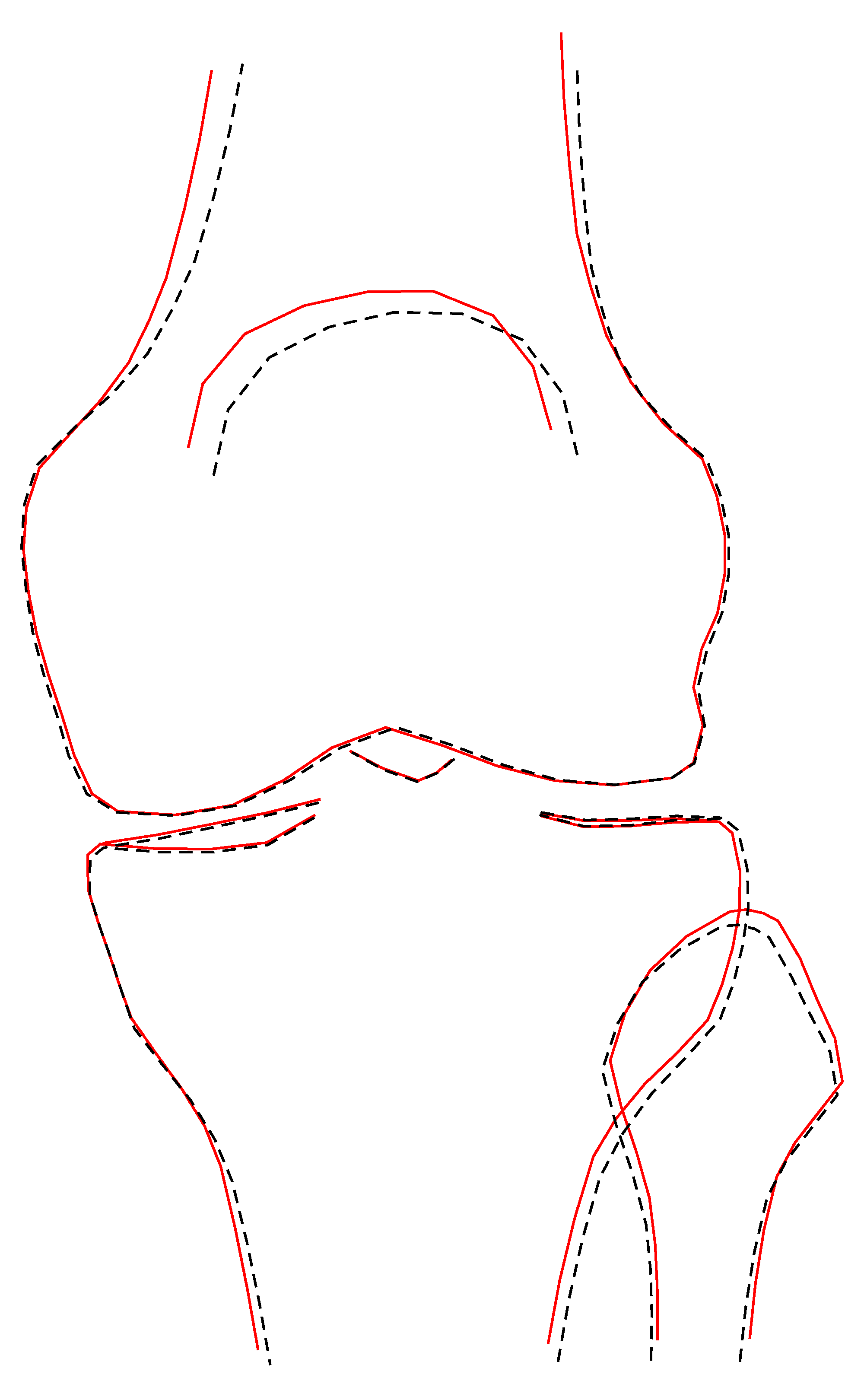

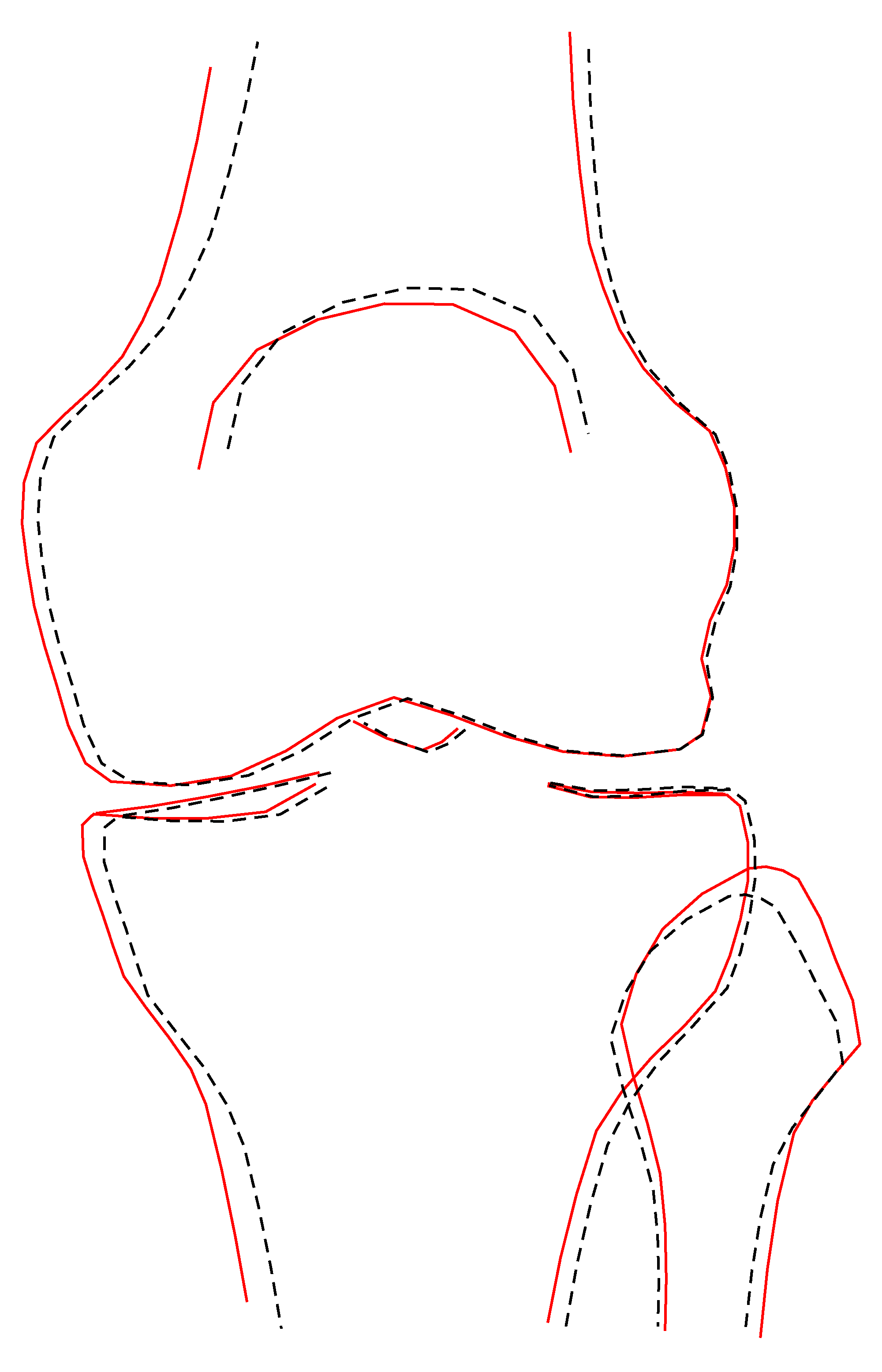

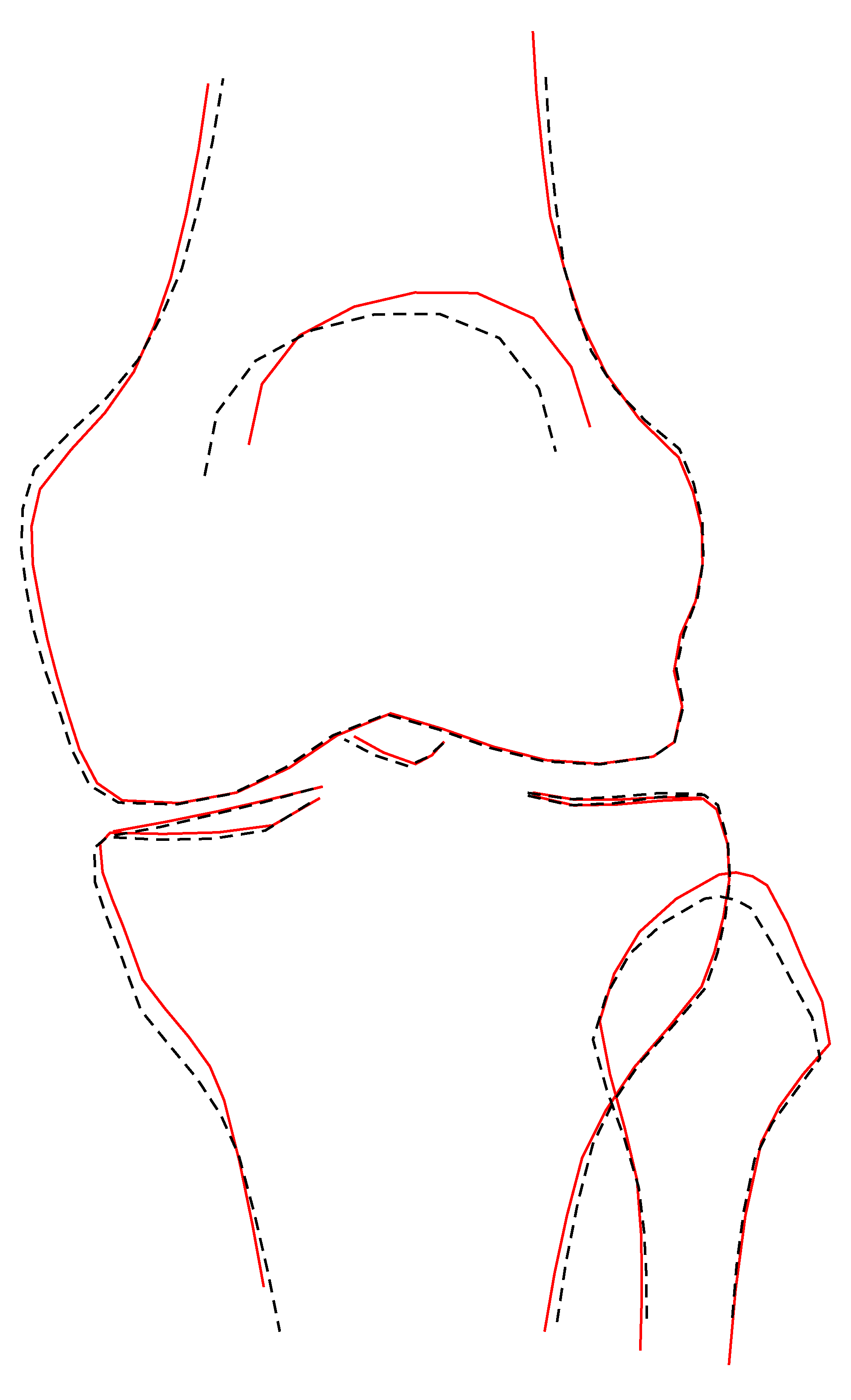


Shapes are aligned at point 17 on the SSM template (corner of the lateral femur).

Grade 1 vs 0

Grade 2 vs 0

Grades 3-4 vs 0


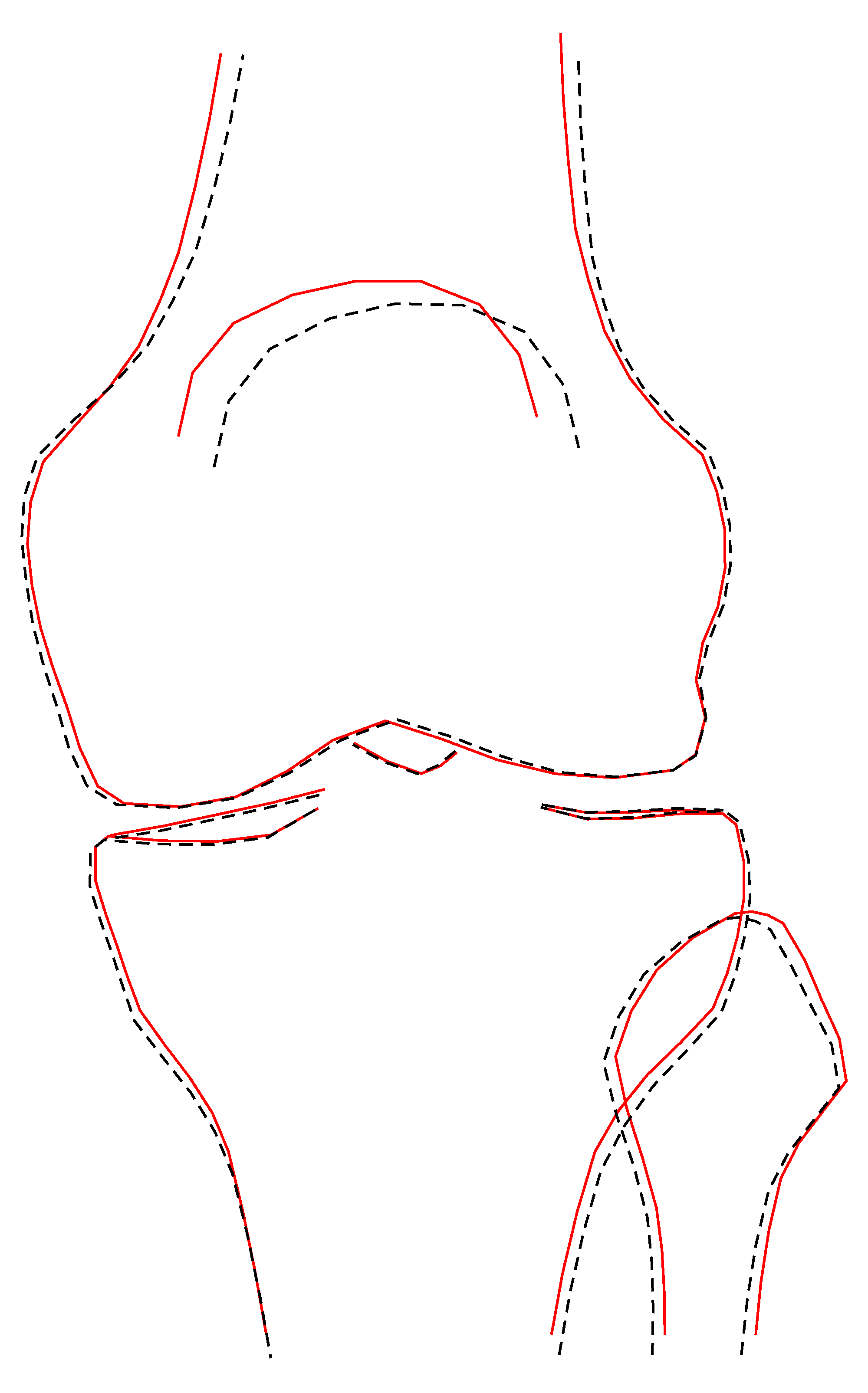

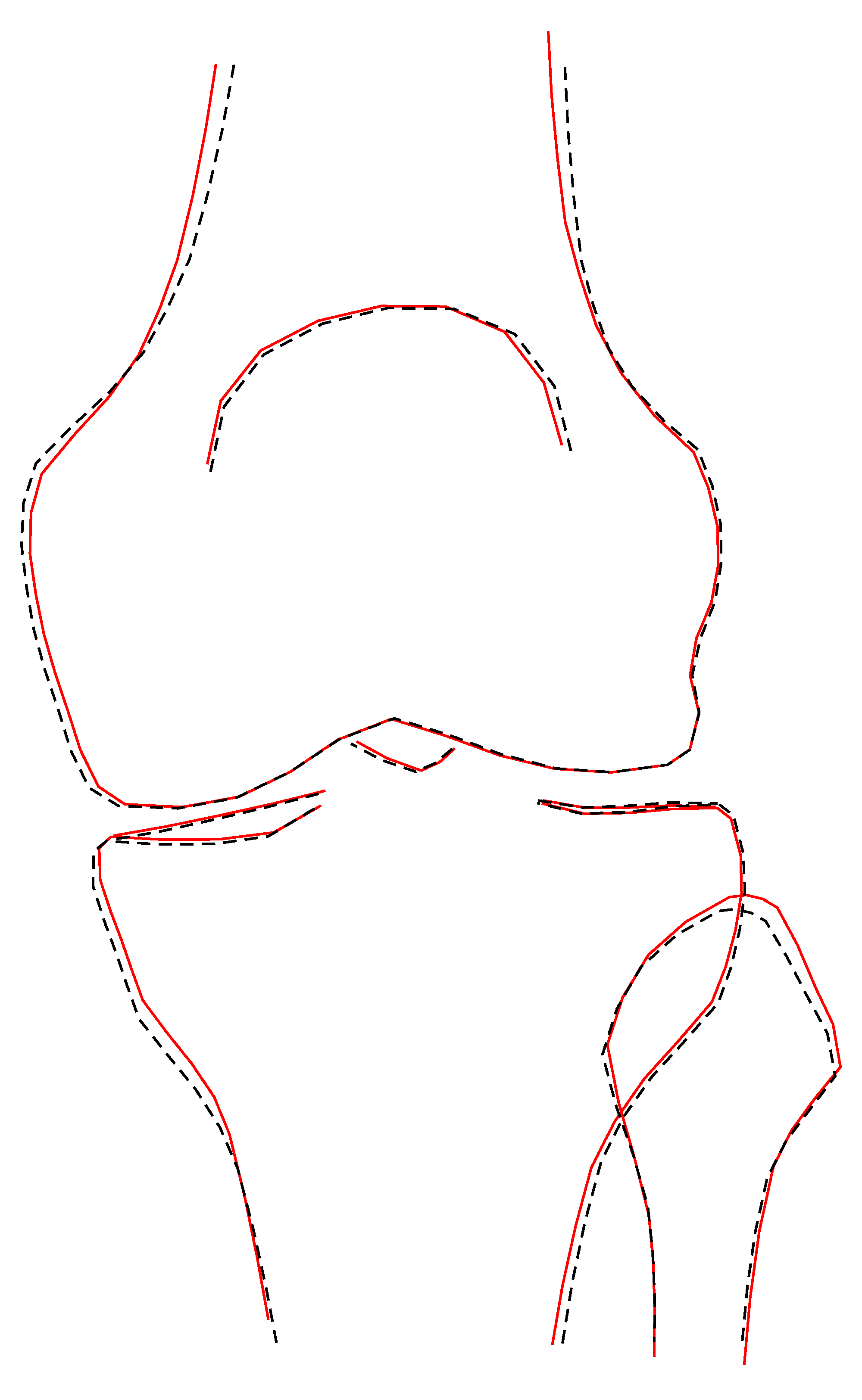

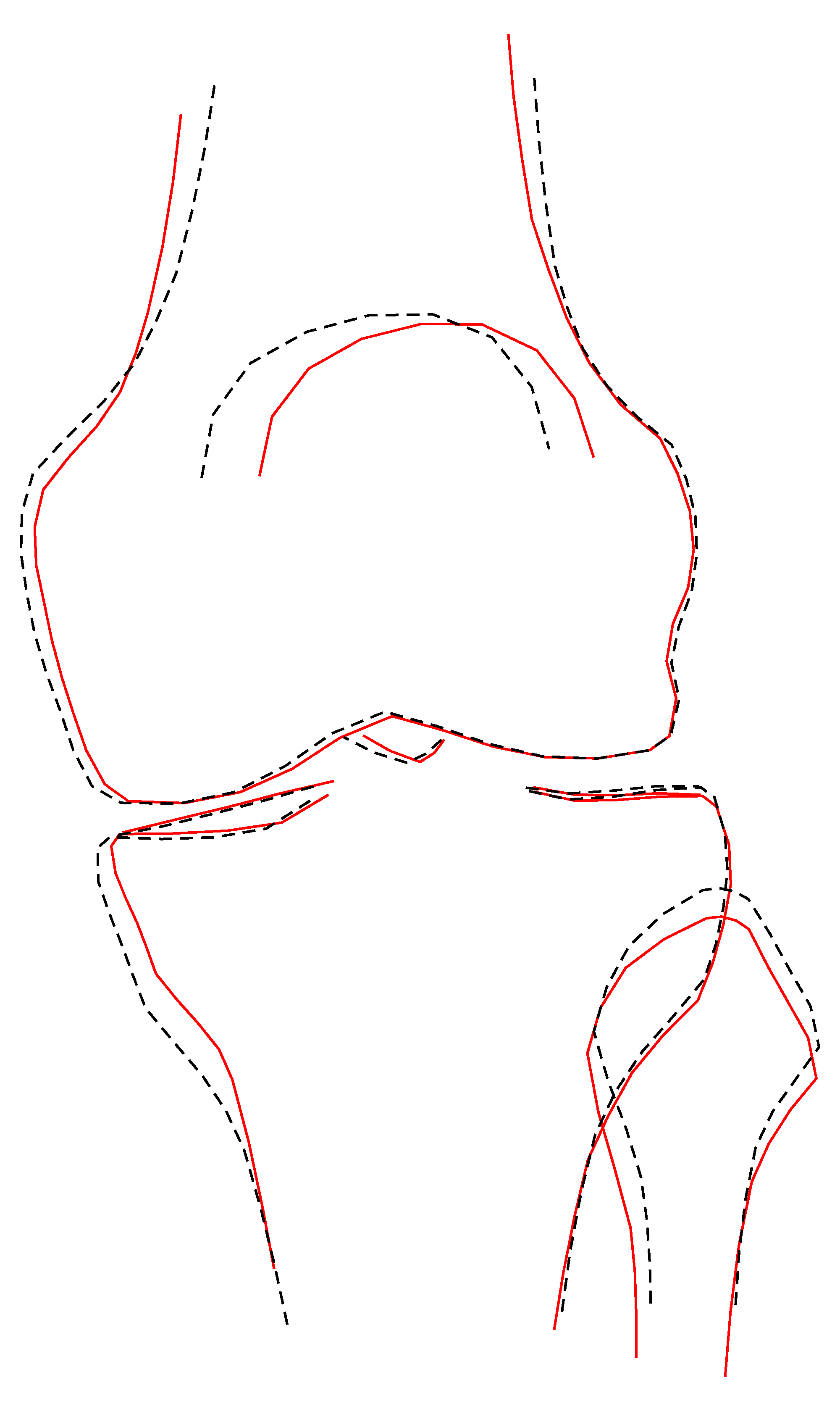


Shapes are aligned at point 17 on the SSM template (corner of the lateral femur).

Supplementary Figure 6: Model 3- adjusted for demographics + HKA angle (female)
